# CSE1L/CAS, the cellular apoptosis susceptibility protein, enhances invasion and metastasis but not proliferation of cancer cells

**DOI:** 10.1186/1756-9966-27-15

**Published:** 2008-07-03

**Authors:** Ching-Fong Liao, Shue-Fen Luo, Li-Tzu Li, Chuang-Yu Lin, Ying-Chun Chen, Ming-Chung Jiang

**Affiliations:** 1 Department of Medical Research, Tungs' Taichung MetroHarbor Hospital, Taichung Taiwan, PR China; 2 Institute of Cellular and Organismic Biology, Academia Sinica, Taipei, Taiwan, PR China; 3 Department of Medicine, Division of Allergy, Immunology and the Rheumatology, Chang Gung Memorial Hospital, Chang Gung University College of Medicine, Tao-yuan, Taiwan, PR China

## Abstract

**Background:**

The cellular apoptosis susceptibility (CAS) protein is regarded as a proliferation-associated protein that associates with tumour proliferation as it associates with microtubule and functions in the mitotic spindle checkpoint. However, there is no any actual experimental study showing CAS (or CSE1 and CSE1L) can increase the proliferation of cancer cells. Previous pathological study has reported that CAS was strongly positive stained in all of the metastasis melanoma that be examined. Thus, CAS may regulate the invasion and metastasis of cancers. CAS is highly expressed in cancers; if CAS is associated with cancer proliferation, then increased CAS expression should be able to increase the proliferation of cancer cells. We studied whether increased CAS expression can increase cancer cell proliferation and whether CAS regulates the invasion of cancer cells.

**Methods:**

We enhanced or reduced CAS expression by transfecting CAS or anti-CAS expression vectors into human MCF-7 breast cancer cells. The proliferations of cells were determined by trypan blue exclusion assay and flow cytometry analysis. Invasion of cancer cells were determined by matrigel-based invasion assay.

**Results:**

Our studies showed that increased CAS expression was unable to enhance cancer cell proliferation. Immunofluorescence showed CAS was distributed in cytoplasm areas near cell membrane and cell protrusions. CAS was localized in cytoplasmic vesicle and immunogold electronmicroscopy showed CAS was located in vesicle membrane. CAS overexpression enhanced matrix metalloproteinase-2 (MMP-2) secretion and cancer cell invasion. Animal experiments showed CAS reduction inhibited the metastasis of B16-F10 melanoma cells by 56% in C57BL/6 mice.

**Conclusion:**

Our results indicate that CAS increases the invasion but not the proliferation of cancer cells. Thus, CAS plus ECM-degradation proteinases may be used as the markers for predicting the advance of tumour metastasis.

## Background

The cellular apoptosis susceptibility (CAS) protein is highly expressed in various cancers including melanomas, lymphomas, breast cancers, hepatomas, ovarian carcinomas, endometrial carcinomas, and colorectal cancers [1-8]. Also, the expression of CAS is correlated positively with high stage and high-grade cancers as well as worse outcome of the cancer patients [1-8]. Thus, CAS may play an important role in regulating cancer development and progression.

*CAS *is the human homologue of the yeast chromosome segregation gene, *CSE1 *[9]. CAS is associated with microtubules and mitotic spindles, the cellular organelles for cell cycle mitosis division; hence CAS is speculated to play a role in cell proliferation and is regarded as a proliferation-associated protein [1,10]. Consequently, many pathological studies demonstrated that the expression of CAS in tumors is related with tumor proliferation in cancer development [2-5]; although there is no any actual experimental study showing CAS can increase cancer proliferation. CAS is highly expressed in cancers; if CAS regulates cancer proliferation during cancer development, then increased CAS expression in cancer cells should be able to increase the proliferation of cancer cells. Instead, our recent study showed that increased CAS expression in human HT-29 colorectal cancer cells inhibited but not stimulated the proliferation of HT-29 cells [11].

Metastatic tumours secrete extracellular matrix (ECM)-degradation proteinases to degrade ECM during invasion. MMP-2 is an ECM-degradation proteinase that secreted from invasive cancer cells and plays an important role in tumor metastasis regulation [12,13]. Tumour cells with strong secretion activities can enhance the secretion of ECM-degradation proteinases and enhance tumour metastasis [14,15]. Experiment showed that MMPs production could be regulated at the level of secretion [16]. Thus, for increasing their invasion and metastasis ability, metastatic tumour cells may develop strong secretory ability to enhance MMPs secretion.

CAS was identified in a study of an antisense DNA fragment that is capable of causing cell resistance to apoptosis induced by bacterial toxins and tumor necrosis factors [17]. CAS also regulates apoptosis induced by cypermethrin [18], interferon-γ [19], and chemotherapeutic drugs including doxorubicin, 5-fluorouracil, tamoxifen, and cisplatin [20]. Pathological studies showed that the expression of CAS was related positively with high stage and high grade of cancers, as well as worse outcome of the patients [1-8]. Notably, a pathological study reported that CAS was strongly positive stained in all of the metastasis melanoma that be examined (n = 23) [2]. Thus, CAS may regulate the invasion and metastasis of cancers.

Tumor metastases are the main characteristics of high grade cancers and are also the main causes of cancer-related mortality. Our recent study also showed that CAS transfection was unable to increase the proliferation of HT-29 cancer cells. Thus, we speculate that CAS regulates invasion and metastasis but not proliferation of cancers. We report here that CAS enhances invasion and metastasis but not proliferation of cancer cells.

## Methods

### Antibodies

Antibodies used in the experiment were mouse anti-CAS monoclonal antibodies (clone 24) (BD Pharmingen Transduction Laboratories, San Diego, CA, USA); rabbit anti-MMP-2 polyclonal antibodies (H-76) (Santa Cruz Biotechnology, Santa Cruz, CA, USA); mouse anti-β-actin monoclonal antibodies (Ab-5) (Neomarker, Westinghouse Drive, CA, USA); goat anti-mouse (or anti-rabbit) IgG secondary antibodies coupled to Alexa Fluor 488 (or Alexa Fluor 568) (Molecular Probes, Eugene, OR, USA); goat anti-mouse IgG secondary antibodies coupled to gold (Jackson ImmunoResearch, West Grove, PA, USA).

### Vectors

We isolated total cellular RNA from HT-29 cells with the Trizol reagent (Invitrogen, Carlsbad, CA, USA). Reverse transcription reaction was carried out using the 1st-strand cDNA synthesis kit (Clontech Laboratories, Palo Alto, CA, USA). The reverse transcription reaction mixture (20 μl) containing 1 μg of DNase-treated total RNA, 20 pmol oligo (dT)_18 _primer, 50 mM Tris-HCl pH 8.3, 75 mM KCl, 3 mM MgCl_2_, 0.5 mM each of dNTP, 1 unit RNase inhibitor, and 200 units/μg RNA of MMLV reverse transcriptase was incubated at 42°C for 1 hour. The PCR reactions were done in a 50-μl reaction mixture containing 5 μl of the reverse transcription reaction mixture, 100 ng each of primer, 0.3 mM Tris-HCl pH 8.0, 1.5 mM KCl, 1 μM EDTA, 1% glycerol, 0.2 mM each of dNTP, and 1 μl of 50×Advantage 2 polymerase mix (Clontech). Primers used to amplify *CAS *cDNA were 5'-TATAGCAATGGAACTCAGCGATGC (sense) and 5'-AGTTTAAAGCAGTGTCACACTGGC (antisense). The DNA was amplified in a GeneAmp PCR System 9700 (Perkin-Elmer, Norwalk, CT. USA) for 35 cycles using the following parameters: 94°C for 30 seconds, 65°C for 30 seconds, and 72°C for 200 seconds with a final extension step at 72°C for 10 minutes. The amplified products were resolved in 1% agarose gel with ethidium bromide. The DNA was eluted and cloned into pGEM-T vector (Promega Corporation, Madison, WI, USA), and was subsequently cloned into the pcDNA3.1 eukaryotic expression vector (Invirogen) to obtain pcDNA-CAS vector. The pcDNA-CAS vector was cut with Apa I and Hind III, and the 516-bp *CAS *fragment (bp 1 to 516) was cloned into the Apa I and Hind III sites of pcDNA3.1 vector in an antisense direction to obtain pcDNA-anti-CAS vector. The identities of the DNA sequences were determined by DNA sequencing.

### Cells and DNA transfections

MCF-7 breast cancer cells, 293 embryo kidney cells, and B16-F10 mouse melanoma cells were from American Type Culture Collection (Manassas, VA, USA). Cultures of cells were as previously described [21]. Cells were transfected with vectors using the Lipofectamine plus reagent (Invitrogen). Transfected cells were selected with a high concentration of G418 for 3 weeks. Multiple drug-resistant colonies (> 100) were pooled together and amplified in mass culture. The transfected cells were maintained in media containing 200 μg/ml G418. For the experiments, cells were cultured in media without G418 and the media were refreshed every 4 days, as MCF-CAS cells are relatively prone to apoptosis in long-time culture when not supplemented with fresh media.

### Immunoblotting

Cells were washed with PBS and harvested by scraping. The harvested cells were washed with PBS and lysed in RIPA buffer (25 mM Tris-HC1 [pH 7.2], 0.1% SDS, 0.1% Triton X-100, 1% sodium deoxycholate, 150 mM NaCl, 1 mM EDTA, 1 mM sodium orthovanadate, 1 mM phenylmethylsulfonyl fluoride, 10 μg/ml aprotinin, and 5 μg/ml leupeptin). The protein concentrations were determined with a BCA protein assay kit (Pierce, Rockford, IL, USA). Fifty micrograms of each protein sample was loaded onto SDS-polyacrylamide gel. Proteins were transferred to nitrocellulose membranes (Amersham Pharmacia, Buckinghamshire, UK). The membrane was blocked at 4°C for overnight with blocking buffer (1% BSA, 50 mM Tris-HCl, pH 7.6, 150 mM NaCl, 0.1% Tween-20). The blots were incubated for 1 hour at room temperature (RT) with primary antibodies followed by incubated with secondary antibodies conjugated to horseradish peroxidase for 1 hour. The levels of protein were detected by enhanced chemiluminescence with an ECL Western blotting detection system (Amersham Pharmacia).

### Cell proliferation assay

Equal numbers of cells (1 × 10^4 ^cells/dish) were seed on 100 mm culture dishes. The media were refreshed every three days. The cell numbers were countered every 24 hours by trypan blue exclusion assays after cell seeding. For each time point, three plates of cells were counted, and each plate was only counted once.

### Flow cytometry analysis

Cells were harvested by 0.1% trypsin-EDTA digestion, washed with PBS containing 0.1% glucose, and fixed in 70% ethanol at 4°C for 16 hours. Cells was stained with a propidium iodide (PI) staining solution containing 100 μg/ml PI, 100 μg/ml RNase A, and 0.1% glucose for 30 minutes. The PI fluorescence was measured with a BD FACS Canto flow cytometer (BD Biosciences, Bedford, MA, USA). A minimum of 10,000 cells in each treatment was analyzed in the flow cytometry analysis.

The proportion of each cell phase was expressed as percentage of the total number of the living cells. The proportion of sub-G1 of each treatment was expressed as percentage of the total number of cells.

### Immunofluorescence

Cells grown on coverslips (12 × 12 mm) were cytospun at 1000 rpm for 10 minutes. Cells were washed with PBS, fixed with 4% paraformaldehyde, permeabilized with 0.1% Triton X-100 in 4% paraformaldehyde, and blocked with PBS containing 0.1% BSA and 0.5% Tween-20. Cells were incubated with primary antibodies, washed with PBS, incubated with goat anti-mouse (or anti-rabbit) IgG secondary antibodies coupled to Alexa Fluor 488 (or 568). Coverslips were examined with a Zeiss Axiovert 200 M inverted fluorescence microscope. Experiments were carried out on duplicate coverslips of three independent experiments and five random fields were imaged per coverslip.

### Immunogold electron microscopy

Cells were washed with PBS and fixed in a mixture of 0.5% glutaraldehyde and 2% paraformaldehyde in Hepes buffer (pH 6.8) for 15 minutes and then in 2% paraformaldehyde in Hepes buffer (pH 6.8) at 4°C for 14 days. Samples were dehydrated with 80% ethanol and infiltrated with increasing concentrations of Lowicryl HM20 resin (Polysciences, Tokyo, Japan). Polymerization of Lowicryl HM20 was performed by UV irradiation (wavelength peak at 360 nm) for 24 hours. Ultrathin sections were cut and then mounted on nickel grids coated with 2% Neoprene (Ohken, Tokyo, Japan). After being sunk in 100% ethanol for 3 minutes, samples were immersed in 0.01 M EDTA (pH 7.2) at 65°C for 24 hours. The samples were washed with PBS three times (5 minutes/wash) and blocked with PBS containing 1% BSA and 0.1% Tween-20 for 15 minutes. The samples were incubated with a mixtures of primary antibodies diluted in PBS (1:30) for 1 hour, washed with PBS three times (5 minutes/wash), reacted with 12-nm gold-labeled secondary antibodies, followed by washing with PBS three times (5 minutes/wash). The samples were stained with uranyl acetate and were examined on a Hitachi H-7000 transmission electron microscope.

### MM-2 secretion analysis

Equal numbers of cells were seed on 100 mm culture dishes. Serum contains high level of endogenous MM-2 and may interfere with the MM-2 secretion assay, thus cells were grown to confluence and than cultured in media without serum supplement for 36 hours. The conditioned media were harvested and the cell numbers were determined. The cell number standardized conditioned media were resolved in 10% SDS-PAGE and the levels of MMP-2 in media were analyzed by immunoblotting with anti-MMP-2 antibodies.

### Matrigel-based invasion assay

Polyvinylpyrrolidone-free polycarbonate filters with 8-μm pore size (Costar, Cambridge, MA, USA) were soaked in matrigel (BD Biosciences) (1:10 in DMEM for transfected B16-F10 cells and 1:50 in DMEM for transfected MCF-7 cells) at 4°C for 36 hours and then incubated at 37°C for 2 hours. The filters were washed 4 times with DMEM and were placed in the microchemotaxis chambers. The cells were treated with 0.1% trypsin-EDTA, re-suspended in DMEM media containing 10% FBS and then washed with serum-free DMEM media. Cells (1 × 10^5^) were finally suspended in DMEM (200 μl) and placed in the upper compartment of the chemotaxis chambers. Culture medium (300 μl) containing 20% FBS was placed in the lower compartment of the chemotaxis chamber to serve as a source of chemoattractants. After being incubated in the cell culture incubator for 10 hours (for transfected B16-F10 cells) or 24 hours (for transfected MCF-7 cells), the cells on the upper surface of the filter were completely wiped away with a cotton swab. The cells on the lower surface of the filter were fixed in methanol, stained with Liu's A and Liu's B reagents, and then counted under a microscope. Cells invaded to the microchemotaxis chambers were also counted. For each replicate, the tumour cells in 10 randomly selected fields were determined, and the counts were averaged.

### Animal metastasis experiment

C57BL/6 mice between 6–7 weeks old (National Laboratory Animal Center, Taipei, Taiwan) were housed in an animal holding room under standard conditions (22°C; 50% humidity; 12-hours light/dark cycle). Each C57BL/6 mouse was injected with viable B16-EV cells or B16-anti-CAS cells (5 × 10^4 ^cells in 50 μl DMEM/mouse) in the tail vein. Each experimental group included 11 B16-EV cells-injected mice and 11 B16-anti-CAS cells-injected mice, and totally 66 mice were used in the experiment. Twenty-five days after injection, the mice were sacrificed and necropsied. The numbers of tumours in lungs were counted by macrography and micrography. Mouse care and experimental procedures were performed following the guideline of the Animal Care Committee of Academia Sinica, Taiwan.

### Statistical analysis

All values are expressed as mean ± standard deviation (SD). Statistical differences were analyzed by two-tailed Student's t-test. The α-level of 0.05 was used to determine statistical significance.

## Results

### Increased CAS expression is unable to enhance the proliferation of MCF-7 cancer cells

**Figure 1:**
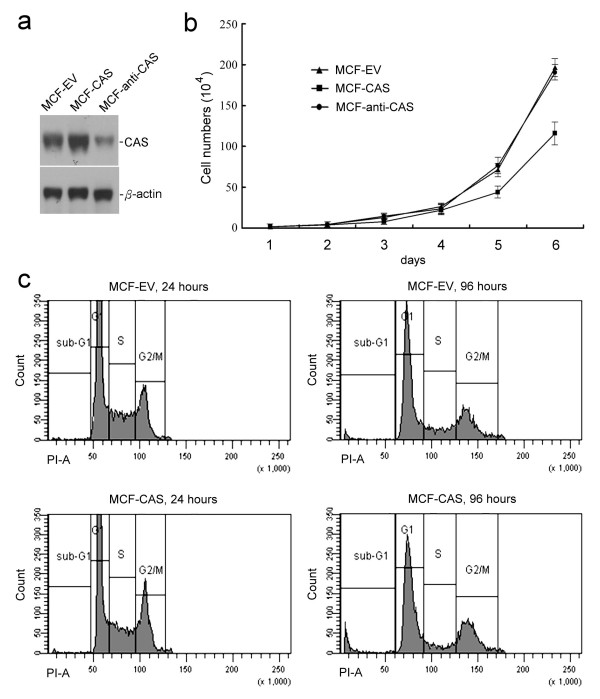
**Increased CAS expression is unable to increase the proliferation of MCF-7 cells**. **(a) **Immunoblotting of CAS expression in MCF-EV, MCF-CAS, and MCF-anti-CAS cells. **(b) **The growth curves of MCF-EV, MCF-CAS, and MCF-anti-CAS cells. The graph represents the results of three independent assays. **(c) **Flow cytometry analyses showed increased CAS expression induced cytotoxicity of MCF-7 cells and decreased cell proliferation. Two experiments with two duplicates were performed and showed similar results; a representative result is shown here. Please see the text for detail.

MCF-7 cells were separately transfected with pcDNA3.1 control vector (EV), pcDNA-CAS vector (CAS), and pcDNA-anti-CAS vector (anti-CAS) to obtain MCF-EV, MCF-CAS, and MCF-anti-CAS cells, respectively (Fig. 1a). For reducing cellular CAS level, antisense DNA method is better than such techniques as gene knockout or siRNA for this study. Because *CAS *is essential for cell survival, knockout of the *CAS *gene or extreme CAS reduction may affect cell survival [22]. Also, CAS was identified in a study of an antisense DNA fragment that is capable of causing cell resistance to apoptosis induced by bacterial toxins [17]; hence reduction of cellular CAS levels by antisense DNA fragment against CAS is sufficient to obtain the cellular effect of CAS reduction. We assayed the proliferations of MCF-EV, MCF-CAS, and MCF-anti-CAS cells to study the effect of CAS expression on the proliferation of MCF-7 cancer cells. CAS is a cellular apoptosis susceptibility protein, thus high CAS expression may be toxic to cells. In the routine cell cultures, we have noted that the growth of MCF-CAS cells was obviously slower than that of MCF-EV and MCF-anti-CAS cells, probably due to the cytotoxicity of CAS. CAS is essential for cell survival, and most MCF-7 cells died after being transfected with the anti-CAS vector (data not shown). However, the growth rate of the established stable cell line, MCF-anti-CAS cells, was similar to the growth rate of the MCF-EV cells (Fig. 1b). On the other hand, the results of cell proliferation assays showed that the growth rate of MCF-CAS cells was indeed slower than that of MCF-EV cells and MCF-anti-CAS cells (Fig. 1b). We speculate that the cytotoxicity of CAS might account for the inhibition of cell proliferation of MCF-CAS cells by CAS. Thus, flow cytometry analyses were performed to study whether CAS overexpression induces cytotoxicity of MCF-7 cells and reduces cell proliferation. MCF-EV and MCF-CAS cells were cultured for 24 or 96 hours and FACS analyses based on DNA content were used to determine the cell cycle distribution. The phase distributions were 56.0% at G1, 22.4% at S, and 21.6% at G2/M for MCF-EV cells cultured for 24 hours; 55.2% at G1, 20.4% at S, and 24.4% at G2/M for MCF-CAS cells cultured for 24 hours; 57.0% at G1, 16.2% at S, and 26.8% at G2/M for MCF-EV cells cultured for 96 hours; 58.2% at G1, 11.3% at S, and 30.5% at G2/M for MCF-CAS cells cultured for 96 hours. The percentages of cells in sub-G1 phase were 1.0% for MCF-EV cells cultured for 24 hours, 1.2% for MCF-CAS cells cultured for 24 hours, 2.0% for MCF-EV cells cultured for 96 hours, and 6.4% for MCF-CAS cells cultured for 96 hours. The sub-G_1 _fraction in a DNA histogram determined by flow cytometry is considered to be the apoptotic cells. The results showed that CAS overexpression (i.e. MCF-CAS cells) increased the percentage of sub-G_1 _fraction of cells (Fig. 1c). The results also showed that CAS overexpression decreased the percentage of MCF-7 cells in S-phase (Fig. 1c). Thus, CAS overexpression induces cytotoxicity of MCF-7 cells and reduces cell proliferation.

### CAS is located in cytoplasm vesicle near cell membrane and cell protrusion

**Figure 2:**
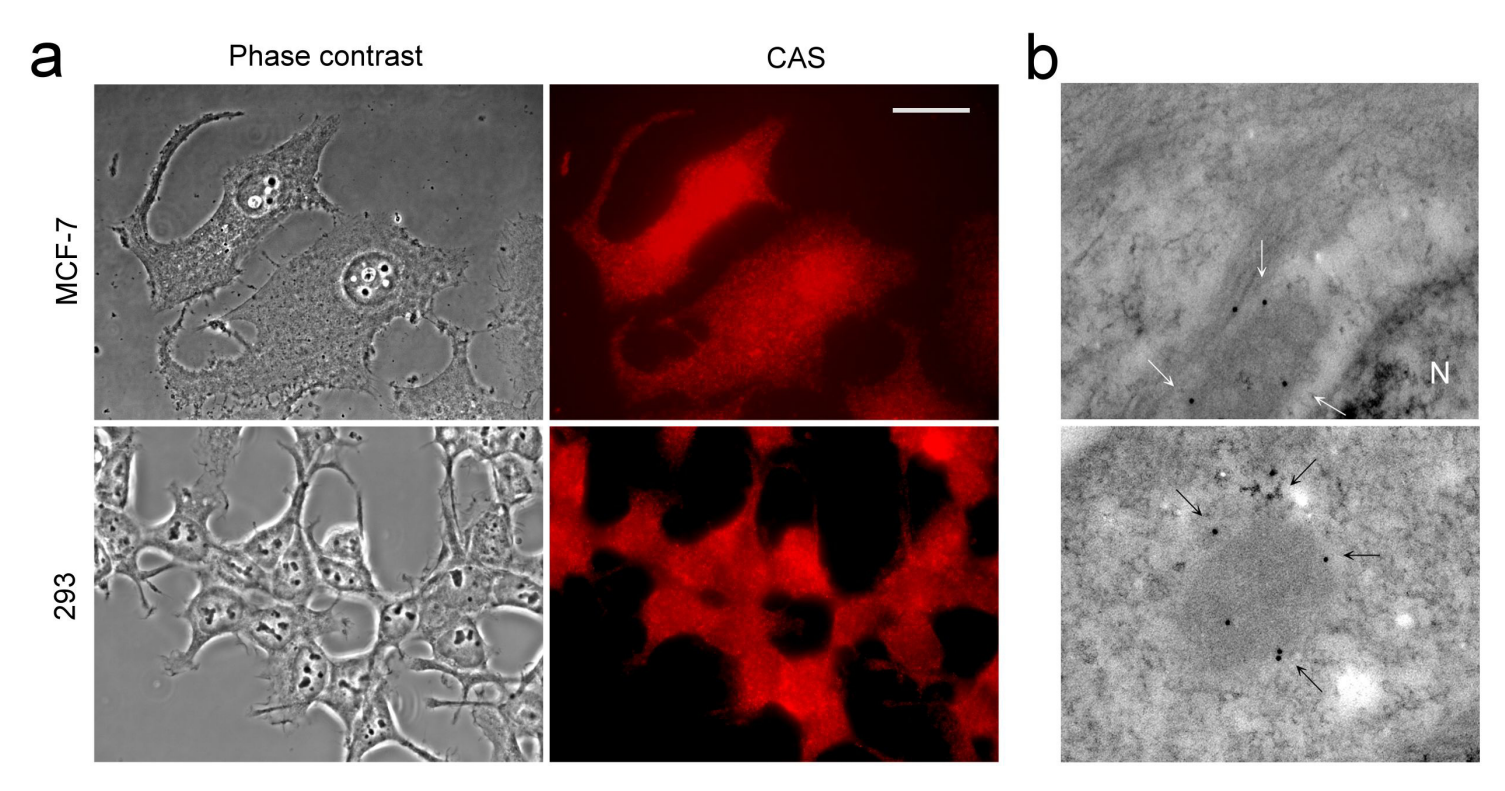
**Immunofluorescence show granular staining pattern of CAS in cytoplasm areas near cell membrane and cell protrusions**. **(a) **Immunofluorescence analyses with anti-CAS antibodies showed punctual staining of CAS in the cytoplasm areas near cell membrane and the cell protrusions of MCF-7 cells and 293 cells. The scale bar represents 20 μm. **(b) **Immunogold electronmicroscopy analyses showed CAS (12-nm gold, arrows) was localized in vesicle and mainly in the vesicle membrane in MCF-7 cells. N indicates nucleus. Original magnification: × 250,000.

Immunofluorescence observations with anti-CAS monoclonal antibodies showed punctual staining of CAS in cytoplasm around perinuclear areas and areas near cell membrane as well as in the cell protrusions of MCF-7 cells and 293 cells (Fig. 2a). CAS binds strongly with importin-α, a nuclear-transport receptor [23], thus CAS around perinuclear areas may mainly associate with the importin-α complex. The punctual staining of CAS in cytoplasm areas near cell membrane and cell protrusion indicates that CAS may be located in cytoplasm vesicles. We studied the location of CAS by electron microscopy. Immunogold electron microscopy analyses showed that CAS (12-nm gold, arrows) was located in vesicle and mainly in the vesicle membrane (Fig. 2b).

### CAS colocalizes with MMP-2

**Figure 3:**
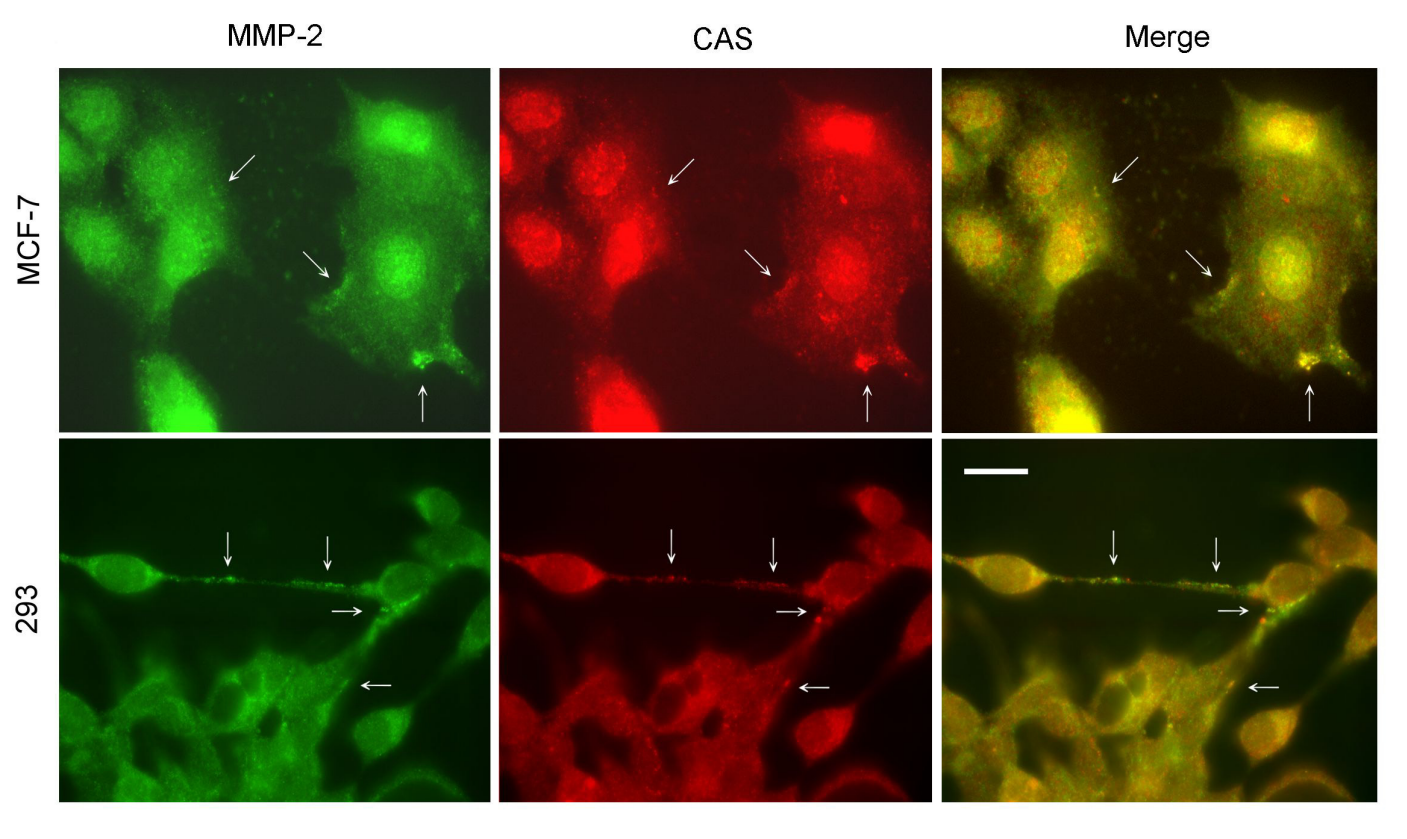
**CAS colocalizes with MMP-2**. Immunofluorescence analyses with anti-CAS and anti-MMP-2 antibodies showed CAS was colocalized with MMP-2 in MCF-7 cells and 293 cells. Arrows indicate some of the colocalzations. The scale bar represents 20 μm.

Our data show that CAS may be located in cytoplasm vesicles. Cytoplasm vesicle plays an important role in regulating cell exocytosis [24]. Thus, CAS may regulate the secretion of tumor cells and hence regulates the invasion and metastasis of cancer cells. MMP-2 plays an important role in regulating tumor metastasis. Our double-stain immunofluorescence studies showed that although not all CAS was colocalized with MMP-2, there were indeed many CAS colocalized with MMP-2 in MCF-7 and 293 cells (Fig. 3).

### CAS regulates MMP-2 distribution

**Figure 4:**
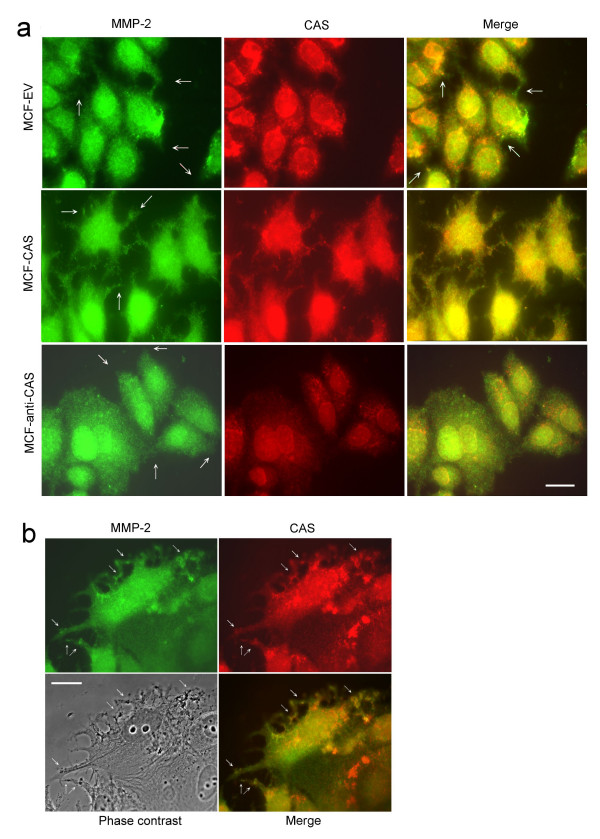
**CAS regulates MMP-2 translocation**. **(a) **Double-stain immunofluorescence analyses of MMP-2 and CAS distributions in MCF-EV, MCF-CAS, and MCF-anti-CAS cells. Please note the increased distribution of MMP-2 in the cell protrusions of MCF-CAS cells. Arrows indicate some of the cell protrusions. The scale bar represents 20 μm. **(b) **Double-stain immunofluorescence showed CAS was colocalized with cytoplasmic vesicle and MMP-2 in MCF-CAS cells. Arrows indicate some of the colocalizations. The scale bar represents 30 μm.

The effects of CAS expression on MMP-2 distribution were studied by double-stain immunofluorescence. In MCF-EV cells, colocalizations of MMP-2 with CAS were mainly observed at cytoplasm areas near cell membrane, and only some in cell protrusions (Fig. 4a). In MCF-CAS cells, many MMP-2 was efficiently translocated to cell protrusions and was obviously colocalized with CAS (Fig. 4a). In our observations, colocalizations of CAS with MMP-2 in MCF-EV cells were only observed in one or two cells per microscopic field. But in MCF-CAS cells, colocalizations of CAS with MMP-2 were observed in most cells especially in the cell protrusions. In a high-resolution photograph, CAS was found to be colocalized with cytoplasmic vesicle and MMP-2 (Fig. 4b).

### CAS regulates MMP-2 secretion and invasion of cancer cells

**Figure 5:**
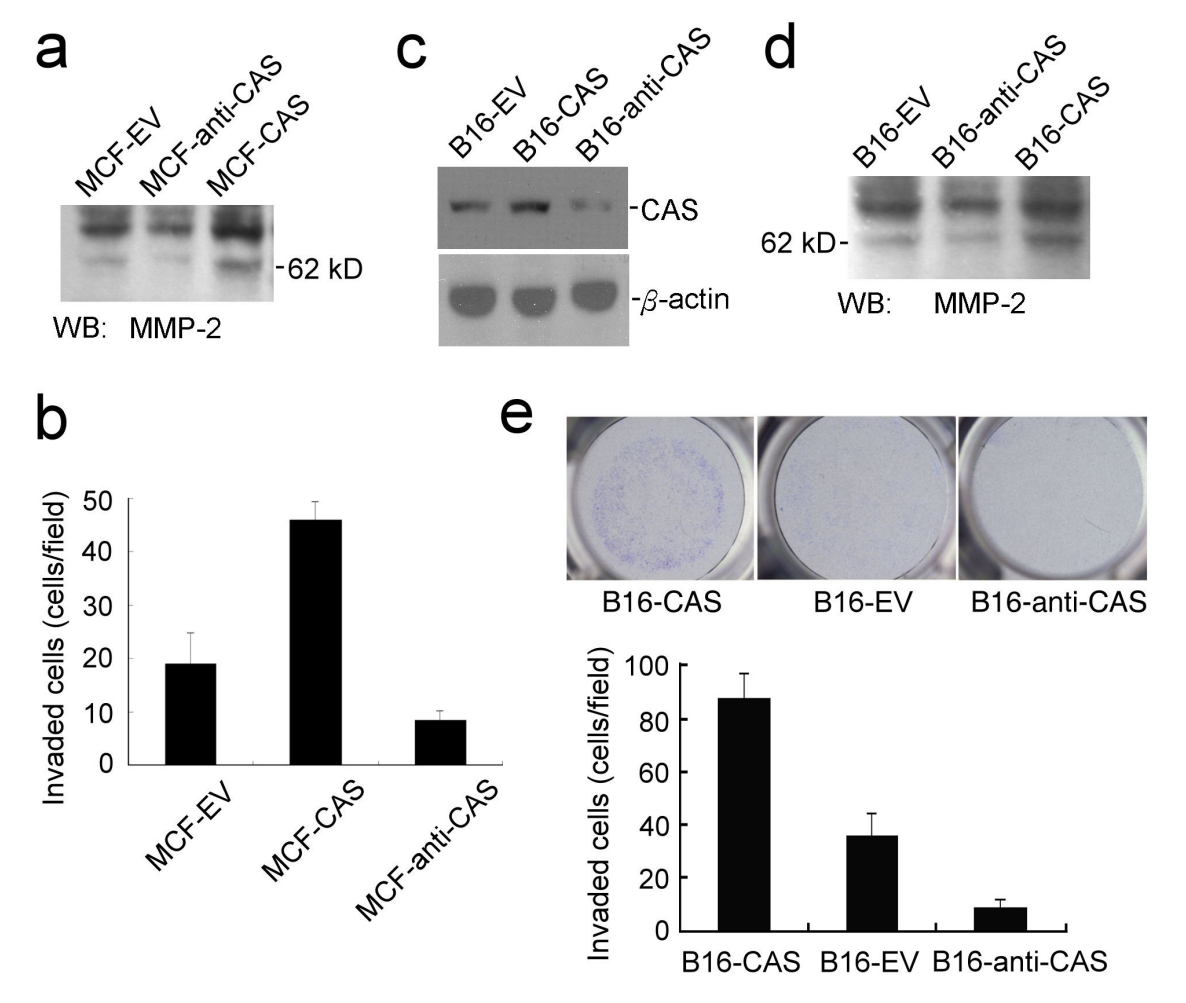
**CAS regulates MMP-2 secretion and invasion of cancer cells**. **(a) **CAS regulates MMP-2 secretion of MCF-7 cells. **(b) **Matrigel-based invasion assays of MCF-EV, MCF-CAS, and MCF-anti-CAS cells. Data represent the means of three independent experiments. **(c) **Immunoblotting of CAS expression in B16-EV, B16-CAS, and B16-anti-CAS cells. **(d) **CAS regulates MMP-2 secretion of B16-F10 cells. **(e) **Matrigel-based invasion assays of B16-CAS, B16-EV, and B16-anti-CAS cells. The upper is a representative photograph of the invaded cells. Data represent the means of three independent experiments.

MCF-EV, MCF-CAS, and MCF-anti-CAS cells were grown to confluence and then cultured in media without serum supplement for 36 hours. The conditioned media were harvested and were subjected to immunoblotting with anti-MMP-2 antibodies. The results showed that MMP-2 secretion from cells was enhanced by increased CAS expression and was reduced by CAS reduction (Fig. 5a). The effects of CAS expression on invasion of MCF-7 cancer cells were assayed by matrigel-based invasion assays. The results showed that increased CAS expression enhanced the invasion of MCF-7 cells by 236.8% (*P *= 0.0024), and reduced CAS expression inhibited the invasion of MCF-7 cells by 57.9% (*P *= 0.0098). The average numbers of the invaded cells were 45.7 ± 4.1, 19.6 ± 6.5, and 8.2 ± 2.1 (cells/field) for MCF-CAS, MCF-EV, and MCF-anti-CAS cells, respectively (Fig. 5b). The secretion of MMP-2 from another tumor cell line, B16-F10 melanoma cells, was also studied. B16-F10 cells were separately transfected with the EV, CAS, and anti-CAS vectors to obtain B16-EV, B16-CAS, and B16-anti-CAS cells, respectively (Fig. 5c). The results of MMP-2 secretion assays also showed that increased CAS expression enhanced MMP-2 secretion, and reduced CAS expression decreased MMP-2 secretion of B16-F10 melanoma cells (Fig. 5d). Matrigel-based invasion assays showed that increased CAS expression enhanced the invasion of B16-F10 cells by 249.2% (*P *= 0.0019), and reduced CAS expression inhibited the invasion of B16-F10 cells by 75.7% (*P *= 0.0073). The average numbers of the invaded cells were 89.5 ± 10.7, 35.9 ± 9.4, and 8.7 ± 2.2 (cells/field) for B16-CAS, B16-EV, and B16-anti-CAS cells, respectively (Fig. 5e). Thus, the results of matrigel-based invasion assay indicate that CAS can regulate the invasion of cancer cells.

### CAS regulates the metastasis of B16-F10 melanoma cells

**Figure 6:**
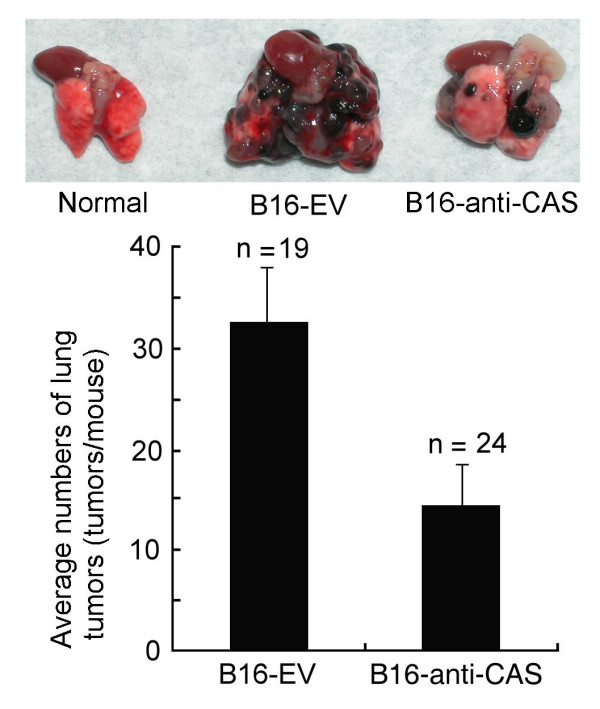
**Animal tumor metastasis experiments show CAS regulates the metastasis of B16-F10 melanoma cells**. Eleven B16-EV cells-injected mice and six B16-anti-CAS cells-injected mice passed away three weeks after injection thus were excluded from the statistics. Six mice (three B16-EV cells-injected mice and three B16-anti-CAS cells-injected mice) didn't grow tumour in lungs and thus were also excluded from the statistics. The results showed that reduced CAS expression inhibited the pulmonary tumour metastasis of B16-F10 melanoma cells in C57BL/6 mice.

Experimental animal tumour metastasis assays were done to study the effect of CAS expression on the metastasis of B16-F10 melanoma cells. B16-F10 cells are high metastatic in C57BL/6 mice, thus we studied whether CAS reduction can reduce the metastasis of B16-F10 cells in C57BL/6 mice. Animal tumour metastasis experiments showed that reduced CAS expression decreased the pulmonary metastasis of B16-F10 cells by 56% in C57BL/6 mice (*P *= 0.0107). The average lung tumour numbers of mice injected with B16-EV cells were 32.7 ± 6.5 tumours/mouse (average tumour diameter 2.6 ± 1.8 mm) and were 14.3 ± 4.6 tumours/mouse (average tumour diameter 2.5 ± 1.5 mm) for mice injected with B16-anti-CAS cells (Fig. 6). Eleven B16-EV cells-injected mice and six B16-anti-CAS cells-injected mice passed away three weeks after injection. Thus, anti-CAS transfection reduced the mortality of mice injected with B16-F10 cells; probably due to anti-CAS transfection reduces the metastasis ability of B16-F10 cells. The results of animal tumour metastasis experiment indicate that CAS can regulate the metastasis of cancers.

## Discussion

CAS is regarded as a proliferation-associated protein that associates with tumour proliferation as it associates with microtubule and functions in the mitotic spindle checkpoint [1-5,10]. But our data showed that CAS overexpression in human MCF-7 cancer cells did not enhance but did reduce the proliferation of MCF-7 cells. The involvement of CAS in proliferation of cancer cells is supported by a study showing that reduction of cellular CAS protein by transfection of antisense cDNA against *CAS *in HeLa cells perturbed progression from G2 (retards transition from G2) to G1 in the cell cycle [25]. CAS may be necessary for M phase mitotic spindle checkpoint in cell cycle progression, but it is quite impossible that tumour cells highly expressing CAS can increase tumour proliferation. The key step that determines the rate limiting for cell proliferation is mainly at the G1-S phase of cell cycle rather than at the mitotic phase [26,27]. Also, CAS is associated with mitotic spindle and regulates the mitotic spindle checkpoint, thus CAS may halt the progression of mitosis until the cells are truly ready to divide. p53 protein also plays a role in activating cell cycle checkpoints, and activation of p53 can stop cell cycle progression at the cell cycle checkpoints [28,29]. The involvement of CAS in proliferation of cancer cells is also supported by a pathological study showing that the expression of the Ki67 proliferation marker in lymphomas was significantly positive correlated with CAS [3]; however this study also reported that a significant fraction of CAS-positive normal and malignant lymphocytes were also found to be Ki-67 negative [3]. In tumours, various oncogenes are activated and various anti-oncogenes are inactivated [30,31], the activated oncogenes and the inactivated anti-oncogenes may stimulate the proliferation of tumours that highly expressing CAS. Thus, positive correlation between CAS and Ki67 expression in tumours is not sufficient to make a conclusion that CAS is related with tumour proliferation. As an apoptosis susceptibility protein, highly expression of CAS can cause the cells susceptible to apoptosis. Our flow cytometry cell cycle study showed that CAS highly expressed in cancer cells can induce cytotoxicity of cells and decreases the proliferation of the cells (Fig. 1).

Formation of cell polarity can stimulate cell-cell adhesion, inhibit tumour migration, and decrease cell proliferation [32]. We have reported that CAS stimulated the polarity of HT-29 cancer cells and thus inhibited the migration of HT-29 cells [11,33]. These results are contradictory to our present report that CAS enhances the metastasis of cancer cells. HT-29 cell line is a special cell line as it is easy to form polarity under in vitro cell culture [34]. In another study, we have observed that CAS was unable to stimulate the polarity of other cancer cell lines including B16-F10, MCF-7, Colon 205, Hep G2, and SK-Hep-1 cancer cells (data not shown). CAS was also unable to increase the proliferation of these cells (data not shown). Thus, the ability of CAS on polarity formation and migration inhibition seems to be a special phenomenon that happened restrictively in HT-29 cell line and the increase of invasion and metastasis should be the correct role of CAS on tumour development.

Although MMPs were previously believed to be synthesized and rapidly secreted, later experiment shows that MMPs are stored in the secretory vesicles in cells and are rapidly secreted in response to angiogentic stimuli [35]. Thus, regulation of the secretion of ECM-degradation proteinases from tumour cells may also plays an important role in regulating tumour metastasis. Our data showed that CAS was localized in vesicle and CAS overexpression enhanced the secretion of MMP-2 and enhanced the invasion and metastasis of tumour cells. Pathological studies also showed that CAS was highly expressed in the metastatic tumours and the expression of CAS was correlated positively with high cancer stage, high cancer grade, and worse outcome of the cancer patients. Taken together, CAS may play an important role in regulating tumour metastasis and CAS plus ECM-degradation proteinases may be used as the markers for predicting the advance of tumour metastasis.

## Conclusion

CAS regulates the invasion but not the proliferation of cancer cells. CAS plus ECM-degradation proteinases may be used as the markers for predicting the advance of tumour metastasis.

## Competing interests

The authors declare that they have no competing interests.

## Authors' contributions

CFL and SFL participated in its design, discussed the results, and helped to draft the manuscript. LTL carried out the immunogold electron microscopy. CYL carried out the immunofluorescence microscopy. YCC carried out the animal metastasis experiment. MCJ conceived of the study, participated in its design, carried out experiments, and wrote the manuscript. All authors read and approved the final manuscript.

## References

[B1] Behrens P, Brinkmann U, Wellmann A (2003). CSE1L/CAS: its role in proliferation and apoptosis. Apoptosis.

[B2] Boni R, Wellmann A, Man YG, Hofbauer G, Brinkmann U (1999). Expression of the proliferation and apoptosis-associated CAS protein in benign and malignant cutaneous melanocytic lesions. Am J Dermatopathol.

[B3] Wellmann A, Krenacs L, Fest T, Scherf U, Pastan I, Raffeld M, Brinkmann U (1997). Localization of the cell proliferation and apoptosis-associated CAS protein in lymphoid neoplasms. Am J Pathol.

[B4] Behrens P, Brinkmann U, Fogt F, Wernert N, Wellmann A (2001). Implication of the proliferation and apoptosis associated CSE1L/CAS gene for breast cancer development. Anticancer Res.

[B5] Wellmann A, Flemming P, Behrens P, Wuppermann K, Lang H, Oldhafer K, Pastan I, Brinkmann U (2001). High expression of the proliferation and apoptosis associated CSE1L/CAS gene in hepatitis and liver neoplasms: correlation with tumor progression. Int J Mol Med.

[B6] Brustmann H (2004). Expression of cellular apoptosis susceptibility protein in serous ovarian carcinoma: a clinicopathologic and immunohistochemical study. Gynecol Oncol.

[B7] Peiro G, Diebold J, Baretton GB, Kimmig R, Lohrs U (2001). Cellular apoptosis susceptibility gene expression in endometrial carcinoma: correlation with Bcl-2, Bax, and caspase-3 expression and outcome. Int J Gynecol Pathol.

[B8] Seiden-Long IM, Brown KR, Shih W, Wigle DA, Radulovich N, Jurisica I, Tsao MS (2006). Transcriptional targets of hepatocyte growth factor signalling and Ki-ras oncogene activation in colorectal cancer. Oncogene.

[B9] Brinkmann U, Brinkmann E, Gallo M, Pastan I (1995). Cloning and characterization of a cellular apoptosis susceptibility gene, the human homologue to the yeast chromosome segregation gene CSE1. Proc Natl Acad Sci USA.

[B10] Scherf U, Pastan I, Willingham MC, Brinkmann U (1996). The human CAS protein which is homologous to the CSE1 yeast chromosome segregation gene product is associated with microtubules and mitotic spindle. Proc Natl Acad Sci USA.

[B11] Jiang MC, Liao CF (2004). CSE1/CAS overexpression inhibits the tumorigenicity of HT-29 colon cancer cells. J Exp Clin Cancer Res.

[B12] Yoon SO, Park SJ, Yun CH, Chung AS (2003). Roles of matrix metalloproteinases in tumor metastasis and angiogenesis. J Biochem Mol Biol.

[B13] Zhang Y, Wang C, Mizukami H, Itoh H, Kusama M, Ozawa K, Jinbu Y (2006). Increased expression and activation of matrix metalloproteinase-2 (MMP-2) in O-1N: hamster oral squamous cell carcinoma with high potential lymph node metastasis. J Exp Clin Cancer Res.

[B14] Moser TL, Young TN, Rodriguez GC, Pizzo SV, Bast RC, Stack MS (1994). Secretion of extracellular matrix-degrading proteinases is increased in epithelial ovarian carcinoma. Int J Cancer.

[B15] Jena BP (2005). Molecular machinery and mechanism of cell secretion. Exp Biol Med (Maywood).

[B16] Taraboletti G, Sonzogni L, Vergani V, Hosseini G, Ceruti R, Ghilardi C, Bastone A, Toschi E, Borsotti P, Scanziani E, Giavazzi R, Pepper MS, Stetler-Stevenson WG, Bani MR (2000). Posttranscriptional stimulation of endothelial cell matrix metalloproteinases 2 and 1 by endothelioma cells. Exp Cell Res.

[B17] Brinkmann U, Brinkmann E, Pastan I (1995). Expression cloning of cDNAs that render cancer cells resistant to Pseudomonas and diphtheria toxin and immunotoxins. Mol Med.

[B18] Izaguirre MF, Vergara MN, Casco VH (2006). CAS role in the brain apoptosis of Bufo arenarum induced by cypermethrin. Biocell.

[B19] Jiang MC, Luo SF, Li LT, Lin CC, Du SY, Lin CY, Hsu YW, Liao CF (2007). Synergic CSE1L/CAS, TNFR-1, and p53 apoptotic pathways in combined interferon-gamma/adriamycin-induced apoptosis of Hep G2 hepatoma cells. J Exp Clin Cancer Res.

[B20] Liao CF, Luo SF, Shen TY, Lin CH, Chien JT, Du SY, Jiang MC (2008). CSE1L/CAS, a microtubule-associated protein, inhibits taxol (paclitaxel)-induced apoptosis but enhances cancer cell apoptosis induced by various chemotherapeutic drugs. BMB reports.

[B21] Jiang MC, Jiang PC, Liao CF, Lee CC (2005). A modified mutation detection method for large-scale cloning of the possible single nucleotide polymorphism sequences. J Biochem Mol Biol.

[B22] Bera TK, Bera J, Brinkmann U, Tessarollo L, Pastan I (2001). Cse1l is essential for early embryonic growth and development. Mol Cell Biol.

[B23] Kutay U, Bischoff FR, Kostka S, Kraft R, Gorlich D (1997). Export of importin alpha from the nucleus is mediated by a specific nuclear transport factor. Cell.

[B24] Pickett JA, Edwardson JM (2006). Compound exocytosis: mechanisms and functional significance. Traffic.

[B25] Ogryzko VV, Brinkmann E, Howard BH, Pastan I, Brinkmann U (1997). Antisense inhibition of CAS, the human homologue of the yeast chromosome segregation gene CSE1, interferes with mitosis in HeLa cells. Biochemistry.

[B26] Moeller SJ, Sheaff RJ (2006). G1 phase: components, conundrums, and context. Results Probl Cell Differ.

[B27] Spugnini EP, Campioni M, D'Avino A, Caruso G, Citro G, Baldi A (2007). Cell-cycle molecules in mesothelioma: an overview. J Exp Clin Cancer Res.

[B28] Giono LE, Manfredi JJ (2006). The p53 tumor suppressor participates in multiple cell cycle checkpoints. J Cell Physiol.

[B29] Ma LL, Sun WJ, Wang Zh, Zh GY, Li P, Fu SB (2006). Effects of silencing of mutant p53 gene in human lung adenocarcinoma cell line Anip973. J Exp Clin Cancer Res.

[B30] Noda H, Mashima R, Kamiyama H, Okada S, Kawamura YJ, Konishi F (2007). Promoter hypermethylation of tumor-related genes in sporadic colorectal cancer in young patients. J Exp Clin Cancer Res.

[B31] Vinodkumar B, Syamala V, Abraham EK, Balakrishnan R, Ankathil R (2007). Germline BRCA1 mutation and survival analysis in familial breast cancer patients in Kerala; South India. J Exp Clin Cancer Res.

[B32] Bilder D (2004). Epithelial polarity and proliferation control: links from the Drosophila neoplastic tumor suppressors. Genes Dev.

[B33] Jiang MC, Liao CF, Tai CC (2002). CAS/CSE 1 stimulates E-cadhrin-dependent cell polarity in HT-29 human colon epithelial cells. Biochem Biophys Res Commu.

[B34] Le Bivic A, Hirn M, Reggio H (1988). HT-29 cells are an in vitro model for the generation of cell polarity in epithelia during embryonic differentiation. Proc Natl Acad Sci USA.

[B35] Nguyen M, Arkell J, Jackson CJ (1998). Active and tissue inhibitor of matrix metalloproteinase-free gelatinase B accumulates within human microvascular endothelial vesicles. J Biol Chem.

